# The effect of perineural dexamethasone on rebound pain after ropivacaine single-injection nerve block: a randomized controlled trial

**DOI:** 10.1186/s12871-021-01267-z

**Published:** 2021-02-12

**Authors:** Jie Fang, Yuncen Shi, Fang Du, Zhanggang Xue, Jing Cang, Changhong Miao, Xiaoguang Zhang

**Affiliations:** 1 Department of Anesthesiology, Zhongshan Hospital, Fudan University, Shanghai, China; 2 Department of Anesthesiology, Jinshan Hospital of Fudan University, Shanghai, China

**Keywords:** Dexamethasone, Nerve block, Rebound pain

## Abstract

**Background:**

Rebound pain after a single-shot nerve block challenges the real benefit of this technique. We aimed to investigate whether perineural dexamethasone addition decreased the incidence of rebound pain after a single-shot nerve block.

**Methods:**

We randomly allocated 132 patients scheduled for open reduction internal fixation of an upper extremity closed fracture under single-shot peripheral nerve block and sedation into two groups. Patients in the dexamethasone group received nerve block with 0.375% ropivacaine and 8 mg dexamethasone, while those in the control group received ropivacaine only. Sixty-three patients in the dexamethasone group and 60 patients in the control group were analyzed for the incidence of rebound pain 48 h after block administration, which was the primary outcome. The secondary outcomes included the highest self-reported numeric rating scale (NRS) pain score, and NRS at 8, 12, 24, and 48 h after the block, sufentanil consumption, sleep quality on the night of surgery, patient satisfaction with the pain therapy, blood glucose at 6 h after the block, pain and paresthesia at 30 days after surgery.

**Results:**

The incidence of rebound pain was significantly lower in the dexamethasone group (7 [11.1%] of 63 patients) than in the control group (28 [48.8%] of 60 patients [RR = 0.238, 95% CI (0.113–0.504), *p* = 0.001]. Dexamethasone decreased opioid consumption in 24 h after surgery (*p* < 0.001) and improved the sleep quality score on the night of surgery (*p* = 0.01) and satisfaction with pain therapy (*p* = 0.001). Multivariate logistic regression analysis showed that only group allocation was associated with the occurrence of rebound pain [OR = 0.062, 95% CI (0.015–0.256)]. Patients in the dexamethasone group reported later onset pain (19.7 ± 6.6 h vs 14.7 ± 4.8 h since block administration, mean ± SD, *p* < 0.001) and lower peak NRS scores [5 (3, 6) vs 8 (5, 9), median (IQR), *p* < 0.001] than those in the control group.

**Conclusions:**

The perineural administration of 8 mg dexamethasone reduces rebound pain after a single-shot nerve block in patients receiving ORIF for an upper limb fracture.

**Trial registration:**

This study was retrospectively registered in the Chinese Clinical Trial Registry (ChiCTR-IPR-17011365) on May 11th, 2017.

**Supplementary Information:**

The online version contains supplementary material available at 10.1186/s12871-021-01267-z.

## Background

Peripheral nerve block (PNB) plays an essential role in anesthesia and multimodal postoperative analgesia in extremity surgeries. It provides multiple benefits, including reducing opioid consumption, better early postoperative pain control and faster hospital discharge [1–3]. However, rebound pain during nerve blocks wear off challenges the real benefit of this technique, especially in ambulatory surgery settings [4]. For patients receiving ambulatory surgery, rebound pain is challenging to prevent or control, especially when it happens at home, which is a common cause of unplanned readmission to the hospital [5]. Initially focused on by orthopedic surgeons, rebound pain after nerve block is gaining increasing attention from anesthesiologists [6–8]. It has been reported after different kinds of surgeries, such as ankle fracture surgery under popliteal sciatic nerve block [9], distal radius fracture fixation under brachial plexus block [10] and shoulder arthroscopy surgery under interscalene brachial plexus block [11]. Understanding the mechanism and searching for strategies to prevent rebound pain is integral to the effective utilization of regional anesthesia.

Uncompliant bridging therapy is supposed to be the main reason for the rebound phenomenon during PNB wear off. However, based on the duration of action of ropivacaine or bupivacaine, the most commonly used local anesthetics for nerve block, there is a chance that the pain burst happens at night when the patient cannot take bridging medicine beforehand. The continuous infusion of local anesthetics through the perineural catheter may reduce the incidence of rebound pain [12]. Nevertheless, perineural catheterization is technically challenging and has the disadvantages of possible dislocation and local infection [13]. Apart from perineural catheter, perineural liposomal bupivacaine holds promise in providing superior pain relief with reduction of postoperative opioids. In 2018, liposomal bupivacaine was approved for interscalene nerve block. VandePitte et al. [14] examined the effects of a mixture of liposomal bupivacaine and standard bupivacaine for interscalene brachial plexus block, compared with standard bupivacaine alone in the setting of multimodal postoperative pain management for major shoulder surgery. The primary outcome measure, worst pain reported by patients, was lower throughout the first week for liposomal bupivacaine, a mean value of 3.6 versus 5.3 on a 0–10 scale. However, opioid consumption, time to first opioid, sleep duration, and adverse effects were not different between the groups. No details of rebound pain was reported in VandePitte’s study. If proven to be safe and effective, adjuvants to local anesthetics could be a useful and economic strategy to prevent rebound pain. The effect of adjuvants to local anesthetics on rebound pain has not yet been thoroughly investigated. Dexamethasone, as a commonly used adjuvant to local anesthetics, prolongs the duration of brachial plexus block without adverse reactions [15]. An K et al. found that perineural, not systemic, dexamethasone added to a clinical concentration of bupivacaine may not only prolong the duration of sensory and motor blockade but also prevent bupivacaine-induced reversible neurotoxicity and short-term “rebound hyperalgesia” in a mouse sciatic nerve block model [16]. To our knowledge, there have been no randomized controlled studies examining the effect of dexamethasone addition on the incidence of rebound pain. We hypothesized that the addition of dexamethasone to ropivacaine could reduce the incidence of rebound pain after single-injection nerve block.

## Methods

This single-center, randomized, double-blind controlled study was approved by the Ethics Committee of Zhongshan Hospital, Fudan University (B2016-079R). Written informed consent was obtained from patients before study enrollment by the investigator. This study followed the Consolidated Standards of Reporting Trials (CONSORT) reporting guidelines.

### Participants

We enrolled patients scheduled for open reduction internal fixation (ORIF) of a closed fracture in the upper extremity under single-shot peripheral nerve block and sedation at Zhongshan Hospital, Fudan University between November 2016 and February 2018. The inclusion criteria were patients aged over 18 years with an ASA physical status of 1 or 2 who had adequate Chinese language skills and a clear understanding of the numerical rating scale (NRS) of pain. The exclusion criteria included patients who refused, those with multiple injuries requiring other surgeries or pain medications, those with preoperative nerve injury, those with a known allergy to ropivacaine or dexamethasone and those with chronic analgesic use. The patients were randomly assigned in a 1:1 ratio to one of two groups (the Dexamethasone and Control groups) according to a computer-generated random number table with SPSS version 23.0 (SPSS Inc., Chicago, Illinois, USA). This allocation was concealed using a sealed opaque envelope that was opened only after the patients were enrolled. Regional medications were prepared by a research nurse who was not involved in the follow-up or care of the patients. The patients, anesthesiologists and outcome assessors were blinded to the group allocation. The physician in charge of generating the allocation sequence and concealment (Fang Du) was not directly involved in the treatment administration or data collection.

### Application of PNB

The patients received ultrasound-guided single-shot nerve block with 40 mL 0.375% ropivacaine and 8 mg dexamethasone in the Dexamethasone group (*n* = 66) and with 40 mL 0.375% ropivacaine alone in the Control group (*n* = 66). Premedication included midazolam 1–2 mg i.v. and fentanyl 50 μg i.v. The same regional anesthetist performed the ultrasound-guided nerve block using a high-frequency linear ultrasound probe. The timing of block administration was recorded. Surgeries for lateral third of clavicle and proximal humeral fractures were performed under combined superficial cervical plexus with interscalene or supraclavicular brachial plexus block. Forearm fracture surgeries involving the musculocutaneous nerve innervation area were performed under axillary brachial plexus block combined with musculocutaneous nerve block in the fascial plane between the biceps and the coracobrachialis muscle. For the combined blocks, 40 mL local anesthetic were divided into 10 mL for the superficial cervical plexus block or musculocutaneous nerve block and 30 mL for the brachial plexus block. The effect of the block was evaluated based on the sensation of pin prick 30 minutes after the block.

### Anesthesia

Standard monitoring was applied, including ECG, BP, HR and SpO_2_. The patients were sedated with dexmedetomidine infusion with a loading dose of 1 μg·kg^− 1^ in 15 min and then at the rate of 0.5 μg·kg^− 1^·h^− 1^ until the surgeon finished the internal fixation. Tropisetron 5 mg i.v. was given at the end of the surgery for postoperative nausea and vomiting prevention. Paracetamol 2 g intravenous (i.v.) drip and parecoxib 40 mg i.v. were given 30 min before the end of surgery.

### Postoperative analgesia protocol

Postoperative multimodal analgesia included patient control intravenous analgesia (PCIA) with 2 μg·h^− 1^ background infusion of sufentanil, 4 μg per bolus, and a lockout time of 6 min as well as parecoxib 40 mg i.v. every 12 h. The patients were instructed to push the self-control button when they felt the numbness of the arm waning. The background infusion was set to maintain a stable blood concentration of sufentanil when rebound pain broke through.

### Outcomes

One of our investigators blinded to the allocation details followed the patients’ pain intensity evaluated by NRS (0–10) at 8, 12, 24, and 48 h after the block and asked them to describe their experience when the block wore off. To avoid interrupting the patients’ night sleep, if the prescheduled follow-up time point fell between 9 pm and 8 am, the patient recorded a pain dairy (Supplement 1) when moderate or severe pain occurred. The other investigators who did not know the group allocation and was not involved in the follow-up decided whether it fit the criteria of rebound pain according to patients’ descriptions. Based on our preliminary observation, we empirically defined rebound pain as severe pain (NRS > 7) that occurs suddenly and cannot be relieved after a PCIA bolus in 30 min; if pain occurs during sleep, it wakes up the patients and makes it difficult for them to go back to sleep. A self-reported sleep questionnaire (Supplement 2), which included six yes-or-no questions, was used to investigate perioperative sleep quality. A score of 1 represents the best sleep, and a score of 6 represents the worst sleep.

The primary outcome of this study was the incidence of rebound pain 48 h after block administration. The secondary outcomes included the highest self-reported NRS score and the hours elapsed since the block administration when the worst pain happened, the pain intensity at 8, 12, 24, 48 h after the block, sufentanil consumption at 24 and 48 h after the block, sleep quality on the night of surgery, patient satisfaction with postoperative pain therapy grading from 1 (strongly unsatisfied) to 5 (strongly satisfied), blood glucose at 6 h after the block, and pain and paresthesia at 30 days after surgery followed by phone call.

### Statistical analysis

The sample size calculation was based on our preliminary observational results that 60% of patients suffered from severe pain (NRS ≥ 7) after ORIF with a single-shot nerve block. We assumed that a 22% decrease in rebound pain incidence with the addition of dexamethasone was clinically significant. With 80% power, 61 patients in each group were required to detect this difference at a significance level of 0.05. We recruited 132 patients with 66 patients per group to compensate for the potential drop out.

Statistical analysis was conducted using SPSS version 23.0 (SPSS Inc., Chicago, Illinois, USA). Continuous data are expressed as the median and interquartile range unless the data was verified to have a normal distribution. The level of significance was set at *p* < 0.05, and 95% confidence intervals were calculated for the primary outcome measures. We performed a preliminary explanatory analysis to examine the relationships between potential covariates and the dependent variable, rebound pain, as well as the independent variable, group allocation. The Mann-Whitney U test was used for the continuous variables, including days after injury, sleep quality score the night before surgery and patients’s satisfactory score. Based on the type of the distribution; chi-square tests were used to assess the associations between the categorical covariates, including surgeon, block type (single or combined approach), fracture location, brachial nerve approach and use of tourniquet. The variance analysis of repeated measurements was used for the NRS socres before and after the surgery.

If the prescheduled follow-up time point fell between 9 pm and 8 am, we used the following data reconciliation strategy for the NRS score: according to the patient’s pain diary, if the follow-up time point was before the first reported pain appeared, the resting and exercise NRS scores at that time point were considered to be 0; if the follow-up time point was later than when the initial pain appeared and earlier than the most severe pain that happened, the NRS score at this point was considered to be the mean value of the first NRS higher than 0 and the highest NRS score (rounded down); if the follow-up time point was later than the most severe pain that happened and the patient described that the pain did not alleviate, the highest pain score was taken as the NRS at that point. If the patient did not describe when the pain was relieved, the data at that time point were considered missing.

## Results

**Fig. 1:**
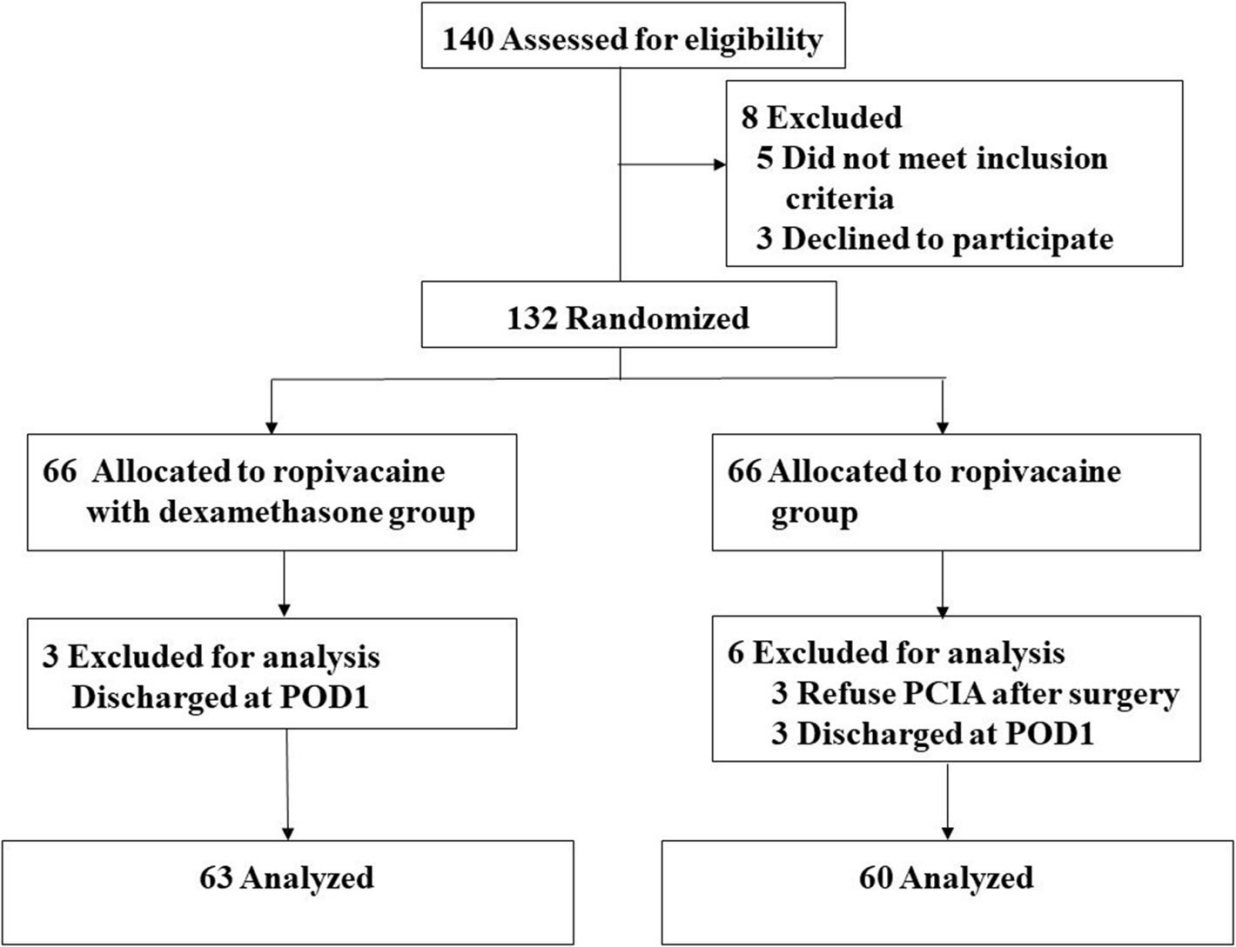
CONSORT diagram of patient recruitment

We assessed 140 patients for eligibility; of these, eight patients declined to participate and were thus excluded. In total, 132 patients were enrolled and randomized. The CONSORT flow diagram is shown in Fig. 1. Three patients in each group were considered lost to follow-up due to being discharged after the surgery without any follow-up data. Three patients in the control group were excluded because they refused to use PCIA immediately after the surgery. Finally, 63 patients in the dexamethasone group and 60 patients in the control group were included in the per-protocol analysis.

The baseline demographic, anesthetic and surgical characteristics of both groups were comparable, with no statistically significant differences (Table 1). The PNBs provided sufficient anesthesia for all operations.

**Table 1:** Demographic, anesthetic and surgical characteristics of the patients. Values are the mean (SD) or number

	Dexamethasone (*n* = 63)	Control (*n* = 60)	*p* value
Age, y	54.6 (17.3)	54.1 (16.1)	0.646
Sex (Male/Female)	23/40	24/36	0.714
ASA classification (I/II)	30/33	29/31	1.000
Time from injury to surgery, days	5.2 (3.1)	6.2 (4.8)	0.08
Fracture location			0.30
Clavicle	6	11	
Proximal humerus	23	24	
Elbow joint	12	5	
Wrist joint	15	11	
Metacarpal	6	8	
Phalange	1	1	
Surgeon			0.95
A	36	36	
B	18	16	
Others	9	8	
Tourniquet (Y/N)	32/31	23/37	0.17
Combined/Single block approach	24/39	32/28	0.09
Brachial nerve block approach			0.062
Interscalene approach	30	33	
Supraclavicular approach	9	15	
Axillary approach	24	12	

Combined approach: combination of interscalene brachial plexus and superficial cervical plexus block or axillary brachial plexus and musculocutaneous nerve block

The incidence of rebound pain was significantly lower in the dexamethasone group [7 (11.1%) of 63 patients] than in the control group [28 (48.8%) of 60 patients]; the relative risk (RR) was 0.238, 95% CI 0.113–0.504, *p* = 0.001.

**Fig. 2:**
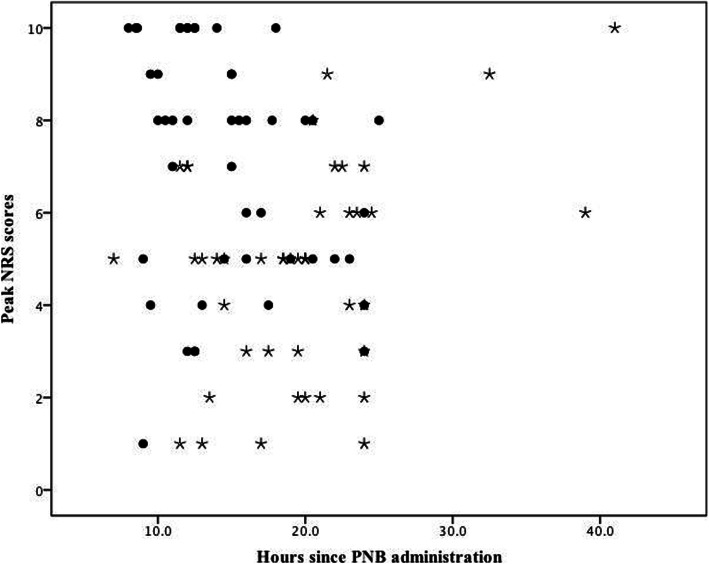
Scatter plots of the highest reported NRS scores in 48 h after block administration in the Dexamethasone group (circles) and the Control group (asterisk)

The patients in the two groups showed different pain profiles when the block wore off. The highest self-reported NRS score and the onset time since PNB administration according to the patients’ pain diary of all the participants are shown in Fig. 2. The highest self-reported pain scores were 5 (3, 6) [median (IQR)] in the dexamethasone group and 8 (5, 9) [median (IQR)] in the control group (*p* < 0.001). The patients in the dexamethasone group reported the highest pain score at 19.7 ± 6.6 h (mean ± SD) since block administration, while those in the control group reported the most severe pain at 14.7 ± 4.8 h (mean ± SD) after block administration.

In univariate analysis, group allocation (*p* = 0.000), block type (single or combination, *p* = 0.042), brachial nerve block approach (*p* = 0.070), usage of tourniquet (*p* = 0.070), NRS pain score with movement (*p* = 0.047) before block administration and sleep quality score on the night before surgery (*p* = 0.066) were found to be significantly associated with rebound pain (marginally significant); thus, we included these factors and fracture location (*p* = 0.430) in multivariate logistic regression analysis. Finally, only group allocation was found to be significantly related to the occurrence of rebound pain (Table 2). Dexamethasone addition into ropivacaine is a protective factor against rebound pain [OR = 0.062, 95% CI (0.015–0.256)].

**Table 2:** Results of multivariate logistic regression analysis for rebound pain

	OR	95%CI	*p value*
Group			
Control	1		
Dexamethasone	0.062	0.015–0.256	**0.000**
Fracture location			
Clavicle	1		
Proximal humerus	2.060	0.404–10.518	0.385
Elbow joint	3.245	0.054–194.966	0.573
Wrist joint	0.364	0.004–35.899	0.666
Metacarpal	0.510	0.004–60.558	0.783
Phalange	1.468	0.007–312.562	0.888
Block approach			
Combined	1		
Single	0.467	0.043–5.101	0.533
Brachial nerve block approach			
Interscalene approach	1		
Supraclavicular approach	0.324	0.025–4.123	0.385
Axillary approach	0.288	0.021–3.911	0.350
Usage of tourniquet			
Y	1		
N	18.423	0.584–581.288	0.098
Preoperative motion NRS			
Pain free (NRS = 0)	1		
Mild pain (NRS 1–3)	3.653	0.268–49.834	0.331
Moderate pain (NRS 4–6)	10.574	0.852–131.189	0.066
Severe pain (NRS 7–10)	6.399	0.508–80.647	0.151
Preoperatibe sleep quality score			
0	1		
1	0.447	0.099–2.006	0.293
2	1.774	0.454–6.933	0.410
3	0.224	0.019–2.615	0.233
4	12.147	0.923–159.781	0.058
5	1.239	0.055–27.847	0.893
6	1.137	0.081–16.050	0.924

**Fig. 3:**
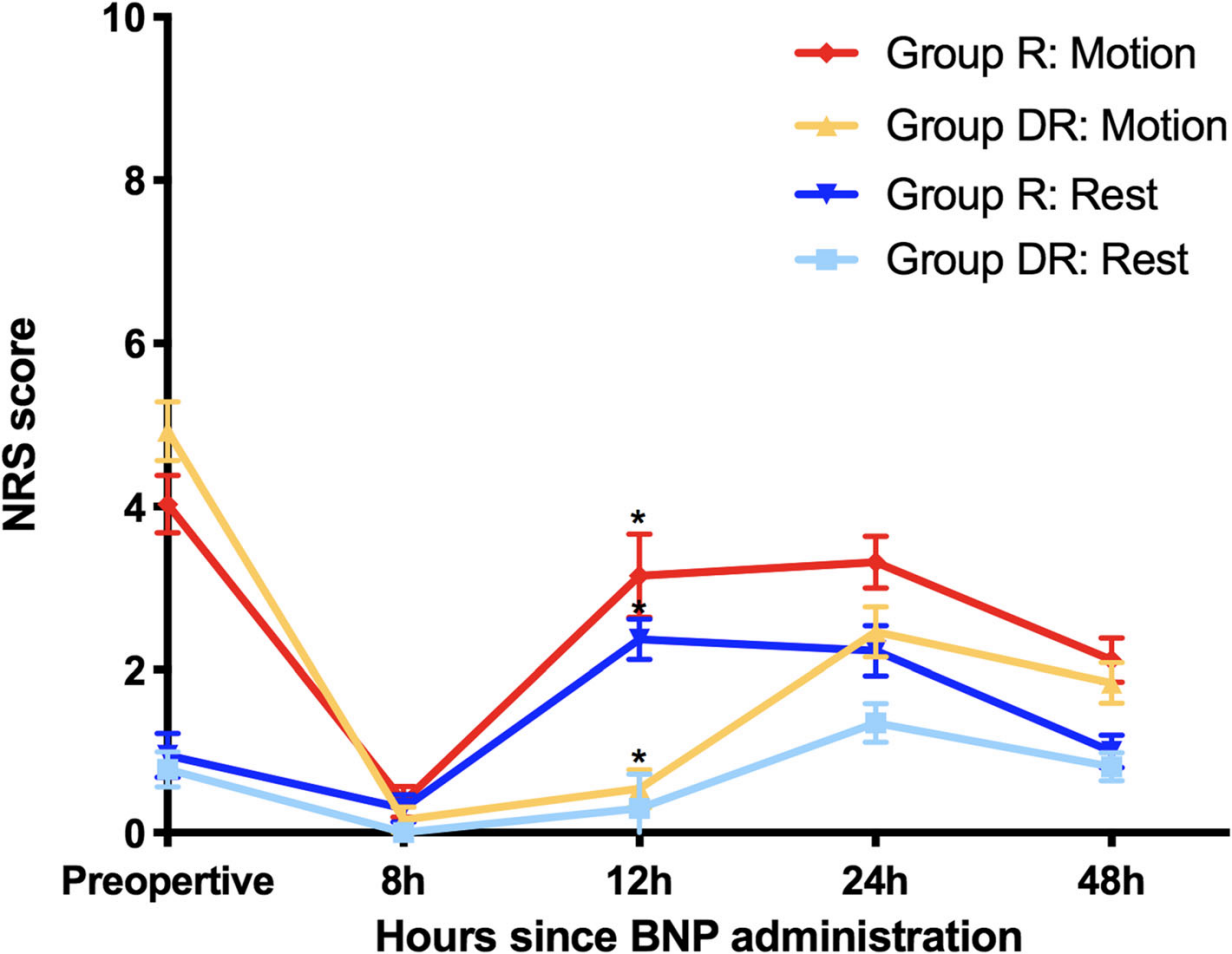
NRS scores before and after the block

There is a significant difference in the trend of NRS scores between the two groups (rest: *p* = 0.004; motion: *p* = 0.038). For the NRS scores at postoperative follow-up time points, a significant difference between the groups existed only at 12 h after block administration, both at rest (*p* = 0.006) and during activity (*p* = 0.001) (Fig. 3).

The cumulative sufentanil consumption and invalid press percentage were significantly lower in patients receiving ropivacaine with dexamethasone than in those without dexamethasone 24 h after the block (*p* < 0.05). No difference was found in opioid consumption between the two groups 48 h after block administration (Table 3).

**Table 3:** Postoperative sufentanil consumption and invalid press of PCA. Values are the mean (SD) and median (IQR)

	Dexamethasone (*n* = 63)	Control (*n* = 60)	*p* value
Sufentanil consumption 24 h, μg	50.49 ± 2.62	66.22 ± 4.05	< 0.001
Sufentanil consumption 48 h, μg	97.76 ± 6.01	108.01 ± 7.13	0.391
Percentage of invalid press 24 h (%)	0.0 (0.0, 22.2)	13.2 (0.0, 29.6)	0.039
Percentage of invalid press 48 h (%)	0.0 (0.0, 20.0)	11.1 (0.0, 29.4)	0.043

We also compared the sleep quality scores and patient satisfaction with pain control therapy and found that patients who received the ropivacaine and dexamethasone combination reported better sleep quality on the night of surgery and more satisfaction with pain therapy (Table 4).

**Table 4:** Sleep quality score and patient satisfaction with pain therapy. Values are expressed as the median (IQR)

	Dexamethasone *n* = 63	Control *n* = 60	*p* value
Sleep score on the night before surgery	1 (0, 3)	1 (0, 2)	0.82
Sleep score on the night of surgery	1 (0, 1)	2 (0, 5)	0.01
Patient satisfaction	5 (4, 5)	4 (2, 5)	0.001

We then investigated whether the addition of dexamethasone caused hyperglycemia. It appeared that patients in both groups showed blood glucose elevation at 8 h after the surgery, but no statistically significant difference was found between the two groups. During the telephone follow-up at 30 days after the surgery, none of the patients complained of paresthesia in the block area or chronic pain at the surgical site.

## Discussion

Perineural dexamethasone is an effective adjuvant used to prolong the duration of the sensory block after regional anesthesia [17]. However, whether it can alleviate the rebound phenomenon after nerve block remains to be investigated. Our study demonstrated that when added to 0.375% ropivacaine, 8 mg of dexamethasone effectively reduced the incidence of rebound pain in patients receiving ORIF for an upper extremity fracture under single-injection nerve block. The addition of dexamethasone not only prolonged the duration of the sensory block but also reduced opioid consumption 24 h after block administration; moreover, it decreased the pain intensity at the point patients described as the most severe pain after the block. Patients who received the addition of perineural dexamethasone reported better sleep quality on the night of surgery and higher satisfaction with postoperative pain therapy.

Rebound pain following single-shot nerve block is a clinically relevant but less valued phenomenon that even diminishes the real benefit of peripheral nerve block in some surgeries [7, 8, 18]. Patients undergoing surgical repair of distal radius fractures experienced different pain profiles after general anesthesia compared with a peripheral nerve block. Although patients with brachial plexus block had less pain immediately after the procedure, 12 h to 24 h later when the block wore off, their pain was greater than that of patients in the general anesthetic group [10].

The mechanism of rebound pain remains poorly understood. The fading of the nerve block is insufficient to explain why there are a certain number of patients who do not experience the outbreak of excruciating pain during the block wear off. It was also noticed that the rebound pain does not respond to intravenous opioid administration [6]. We found that although we provided a background infusion of a high lipid-soluble opioid, sufentanil, to maintain a steady blood concentration and a relatively short lockout time of 6 min in PCA, the patients who suffered from rebound pain were still not relieved by PCA administration. In Williams’ study [19], some patients described rebound pain as an intense burning pain initially as the nerve block resolves. This evidence might suggest a neuropathic instead of a nociceptive component of rebound pain after nerve block. Kolarczyk’s study on rats [20] found that 0.5% ropivacaine induced transient heat hyperalgesia in the setting of resolved mechanical analgesia. Early studies have also suggested that local anesthetics can cause nerve swelling and alter the permeability of the outer membrane of the nerve, leading to abnormal nerve conduction [15]. Therefore, local anesthetic toxicity and the proinflammatory effect of local anesthetics [21] might contribute to the occurrence of rebound pain.

Dexamethasone is a highly potent long-acting glucocorticoid. It improves the quality and prolongs the duration of PNB over LA alone [22]. The mechanism is not fully understood, but it has been suggested that the possible mechanism includes attenuating the release of inflammatory mediators, reducing ectopic neuronal discharge, and inhibiting the potassium channel-mediated discharge of nociceptive C-fibers [23–25]. K et al. found that perineural dexamethasone added to a clinical concentration of bupivacaine prevented bupivacaine-induced reversible neurotoxicity and short-term “rebound hyperalgesia” in a mouse sciatic nerve block model [16]. We demonstrated that adding 8 mg dexamethasone to 0.375% ropivacaine reduced the incidence of rebound pain from 48.8 to 11.1% after ORIF of upper extremity fracture under single-shot nerve block.

In Brian Williams’s retrospective study [26] on additives to a single-injection nerve block, the addition of 2 mg of perineural dexamethasone led to a more favorable rebound pain profile than “other than 2 mg” doses (i.e., no dexamethasone or 4 mg total perineural dexamethasone). According to the systemic review, the dose of dexamethasone utilized in the perineural administration ranged from 4 mg to 10 mg [27]. We chose 8 mg because it is a commonly selected dose in studies on the effect of dexamethasone as an adjuvant to local anesthetics. However, a more recent systematic review from Kirkham KR. showed that there is currently very low quality evidence that 4 mg of perineural dexamethasone represents a ceiling dose that prolongs analgesia duration [28]. Whether a lower dose of dexamethasone provides the same or better rebound pain profile needs to be investigated, especially in some populations, such as diabetic patients.

Rune S et al. prospectively followed 21 patients scheduled for acute open reduction, and the internal fixation rebound phenomenon was less pronounced in patients older than 60 years, whereas most of them suffered from moderate pain (NRS 4–6) during the block effect wear off [8]. We did not find an association between rebound pain incidence and age in the correlation study. However, if grouping the patients by > 60 years or ≤ 60 years, the patients younger than 60 years had a relatively higher incidence of rebound pain (31% vs 23%). Whether the pain trajectory after nerve block changes gradually with aging or displays a drastic change at a certain age needs to be clarified in future studies.

It is challenging to define rebound pain after nerve block. Williams BA et al. [18] described rebound pain scores as a quantifiable difference between the highest NRS score after the nerve block wore off and the last NRS score when the nerve block was still providing pain relief. From our preliminary results, under most circumstances, rebound pain happens all of a sudden, either at night or elicited by movements. Based on the findings from our preliminary observational study, we empirically defined rebound pain as “severe pain (NRS>7) that breaks out in 48 hours after single-shot nerve block, whether at rest or elicited by movement, and cannot be relieved by multiple PCIA boluses in 30 minutes; if the pain occurs during sleep, it wakes up the patients and makes it difficult for them to go back to sleep”. Lavand’homme P [18] provided a detailed description of the definition and characteristics of rebound pain. Our definition is similar to that of Lavand’homme P except that we extend the time limit to 48 h because dexamethasone addition elongates the block duration. We failed to find a unique description of the pain characteristic as burning or aching, so we eliminated the pain characteristic as one of the criteria. Since the rebound phenomenon is hard to detect during regular follow-up, we gave the diagnosis based on the patients’ self-report and pain diary. Further studies are needed to unify the definition of rebound pain to facilitate more randomized trials in this area.

Although dexamethasone is one of the most common additives to a nerve block, there are still some safety concerns. Desmet [29] reported an increase in blood glucose concentrations in the group treated with dexamethasone, which needed insulin therapy. We found that the blood glucose at 6 h after the surgery increased, but there was no significant difference between the two groups, and none of our patients required insulin therapy. This might be because we ruled out patients with diabetes.

There are limitations to our study. We did not investigate the effect of intravenous dexamethasone on rebound pain occurrence. Therefore, we cannot determine whether the reduction in the rebound phenomenon is due to perineural or systemic effects from the absorption of dexamethasone. The comparison of intravenous and perineural dexamethasone showed conflicting results regarding whether the prolongation of the nerve block is of systemic or perineural origin. The results from a meta-analysis showed that for bupivacaine, the addition of perineural dexamethasone leads to a statistically significant prolongation of analgesic duration by 21% compared with intravenous administration. For ropivacaine, the mean duration of analgesia was increased by 12% with perineural dexamethasone compared with systemic dexamethasone, which did not reach statistical significance. The author concluded that the finding of equivalence between both routes of administration remains underpowered for ropivacaine, and a total of 1124 patients would be needed before suggesting a definitive conclusion [30]. Because intravenous dexamethasone has a strong anti-inflammatory effect and perineural administration is still off-label, further studies are needed to investigate the effect of intravenous dexamethasone on the incidence of rebound pain.

## Conclusion

This single-center, randomized, double-blind controlled study revealed that the addition of 8 mg dexamethasone to ropivacaine provides the benefit of reducing rebound pain after a single-shot nerve block in patients receiving ORIF for an upper limb fracture. It also reduces opioid consumption 48 h after the block and decreases the pain intensity at the point patients describe as the most severe pain after the block. Dexamethasone addition improves sleep quality on the night of surgery and patients’ satisfaction with postoperative pain therapy.

## Supplementary Information

**Additional file 1.**

**Additional file 2.**

## Data Availability

The dataset supporting the conclusions of this article is available in Mendeley Data, Zhang, Xiaoguang; Cang, Jing; Du, Fang; Xue, Zhanggang; Shi, Yuncen; Fang, Jie (2020), “Data for: The effect of perineural dexamethasone on rebound pain after ropivacaine single-injection nerve block: A randomized controlled trial”, Mendeley Data, V1, doi: 10.17632/mvy4tjfdcj.1.

## Abbreviations

PNB: Peripheral nerve block
ORIF: Open reduction internal fixation
PONV: Postoperative nausea and vomiting
PCIA: Patient control intravenous analgesia
