# Supplementary material for: The effect of perineural dexamethasone on rebound pain after ropivacaine single-injection nerve block: a randomized controlled trial
Source: BMC Anesthesiol. 2021 Feb 12;21:47. doi: 10.1186/s12871-021-01267-z (PMC7879628; doi:10.1186/s12871-021-01267-z)
Supplement: Supplementary file 1 — Additional file 1. [file 12871_2021_1267_MOESM1_ESM.docx]

Pain Dairy

Name: ID:

Date of surgery:

POD

|  | Start feeling pain | If the pain get worse，please record | | | | |
| --- | --- | --- | --- | --- | --- | --- |
|  |  | 1 | 2 | 3 | 4 | 5 |
| Time |  |  |  |  |  |  |
| NRS score (0-10) |  |  |  |  |  |  |
| Relate to movement | Yes / No |  |  |  |  |  |
| Difficult to go back sleep | Yes / No |  |  |  |  |  |
| NRS score (0-10) 30min after PCA |  |  |  |  |  |  |
| Use Analgesics other than PCIA |  |  |  |  |  |  |

Please describe your pain and tick the most related description fitted to your situation

| Feeling | |
| --- | --- |
| Throbbing |  |
| Shooting |  |
| Stabbing |  |
| Sharp |  |
| Cramping |  |
| Gnawing |  |
| Hot-burning |  |
| Aching |  |
| Heavy |  |
| Tender |  |
| Splitting |  |
| Feeling related emotion | |
| Tiring-exhausting |  |
| Sickening |  |
| Fearful |  |
| Punishing-cruel |  |
