# Supplementary material for: The effect of perineural dexamethasone on rebound pain after ropivacaine single-injection nerve block: a randomized controlled trial
Source: BMC Anesthesiol. 2021 Feb 12;21:47. doi: 10.1186/s12871-021-01267-z (PMC7879628; doi:10.1186/s12871-021-01267-z)
Supplement: Supplementary file 2 — Additional file 2. [file 12871_2021_1267_MOESM2_ESM.docx]

Sleep quality score

|  | POD 0 | POD 1 |
| --- | --- | --- |
| Did you have difficulties in falling asleep last night? |  |  |
| Did you sleep less than 3 hours last night? |  |  |
| Did you wake up frequently last night? |  |  |
| Did you wake up because of the pain? |  |  |
| Was it difficult to fall asleep again after waking up? |  |  |
| Do you feel tired during the daytime? |  |  |

*Yes=1 No=0
